# Third Dentition

**Published:** 1847-10

**Authors:** J. Sanford

**Affiliations:** Dentist, Chillicothe, Ohio


The present and interesting case of Third Dentition, is from our friend Joseph Sanford, Dentist, of Chillicothe, Ohio. But few cases of the kind are recorded, and it is interesting, from the fact that it shows the powerful effort sometimes made by nature to repair the ravages made on the system by disease. Tis true, cases are on record, where she has more successfully repaired the breach than in the present instance—for Good, in his work on Neuerology, gives one or two cases, where the teeth were perfect and had their usual connection with the jaw, and consequently were of service to the individual. I do not recollect of any case out of some five or six recorded, that occurred where the person was under the age of fifty or even sixty years of age.

Dr. Sanford very kindly forwarded me a specimen of these teeth, which have been deposited in the museum of the Ohio College of Dental Surgery. We hope the Profession will more generally adopt the practice of recording all anomalous cases that occur in practice.

We give the etter entire, for it gives a short and concise history of the case.—Cin. Ed.

### *Case of Third Dentition, by* JOSEPH SANFORD, *Dentist.*

CHILLICOTHE, OHIO.

PROFESSOR JAMES TAYLOR:

*Dear Sir*—The case of Third Dentition, which has come under my observation, differs, in some respects, from any that I have seen on record. The subject is a widow, aged eighty-two. When in her sixtieth year she lost her last tooth. The above is confirmed by one of her daughters. So from their statement there was a lapse of twenty-two years intervening between her second and third sets of teeth. I have paid some attention to this old lady through her third dentition, and find but one tooth which is anything like perfection—that is the one I sent you. Ten teeth in all have made their appearance. Two lower incissors, two upper front incissors, four bicuspides above right and left, two lower bicuspides left side. These teeth, with the one exception, are small pieces of brown bone when cut, and act as foreign bodies, which become very painful to the patient, and have to be extracted as fast as cut.

The one which was perfectly formed was very white when first extracted, but has since turned brown as you will perceive.

Respectfully, yours, &c.,

JOSEPH SANFORD.

